# Knowledge, Attitude, and Practices regarding Obesity among Population of Urban (Douala) and Rural (Manjo) Areas in Cameroon

**DOI:** 10.1155/2023/5616856

**Published:** 2023-08-19

**Authors:** Fabrice Fabien Dongho Dongmo, William Djeukeu Asongni, Aymar Rodrigue Fogang Mba, Rebecca Madeleine Ebelle Etame, Diana Ngo Hagbe, Guileine Linda Dongho Zongning, Suzie Vanissa Nkepndep Touohou, Marie Modestine Kana Sop, Rosalie Annie Ngono Ngane, Inocent Gouado

**Affiliations:** ^1^Department of Biochemistry, Faculty of Science, University of Douala, P.O. Box 24157, Douala, Cameroon; ^2^Department of Home Economic, Advanced Teacher's Training College for Technical Education, University of Douala, P.O. Box 1872, Douala, Cameroon

## Abstract

Knowledge, attitude, and practice (KAP) studies have recently been suggested as a useful tool to understand the specificity of the population related to a disease. However, in Cameroon, there is a lack of information based on KAP studies regarding obesity. This study has been designed to collect basic indicators on the KAP of the populations regarding overweight and obesity in urban and rural areas in Cameroon (Douala and Manjo). For this purpose, an epidemiological community-based cross-sectional descriptive study was conducted in these two areas using a well-structured questionnaire. Sociodemographic and medical characteristics and KAP information were assessed. For the quantification of KAP, a score varying from 0 (poor knowledge, attitude, or practices) to 100 (good knowledge, attitude, or practices) was attributed for each question. Correlations between knowledge, attitude, and practice were determined using inferential statistics tests which were *χ*^2^ test, independent Student *t*-test, ANOVA (followed by Tukey's post hoc test), and Pearson correlation coefficient. Results reveal that living in a rural area (Manjo), being overweight or obese, having complete secondary education, and being married increase the knowledge and the practice score. There is a strong and positive correlation between knowledge and practice score. However, there is no association between attitude and practice and between attitude and knowledge. Reducing the disparities between knowledge, attitude, and practices constitutes a serious track in a holistic strategy for the management of obesity in these areas.

## 1. Introduction

The prevalence of obesity is growing worldwide, and since 1975, it has increased by 300% [1]. Indeed, in 2016, there were 2 billion (39%) overweight people and 650 million (13%) obese people worldwide. In 2020, globally, 39 million children under the age of 5 and more than 340 million aged of 5-19 were overweight and obese [2]. In Cameroon, Tchoubi et al. [3] have reported a prevalence of obesity and overweight of 8% among children aged 6 to 59 months in Cameroon in 2011, while Wamba et al. [4] noted in children of 8-15 years old at Douala a prevalence of 14.3% in 2010. Concerning adults, Engle-Stone et al. [5] reported a prevalence of obesity and overweight of 22-55.5% among women. Also, Biyegue et al. [6] found in Douala town a prevalence of obesity and overweight of 54.2% among adults of both sexes in 2016, while Simo et al. [7] noted a prevalence of 50.1% among adults of both sexes in West region in 2018.

Obesity is characterized by an extra amount of adipose tissue at a degree that influences the physiological, psychological, and physical health and well-being of an individual [8]. Obesity is associated with an increased risk of many other diseases and disorders, including cardiovascular diseases, kidney diseases, diabetes, certain cancers, inflammatory diseases, infertility, immune system dysfunction, and infections [8–10]. Besides, there is an association between obesity, severity, and mortality in patients with infectious diseases [11] such as COVID-19.

Various risk factors are associated with obesity, including biological factors, environmental factors, and behavioral factors. Biological factors include genetics, brain-gut axis, hereditary character, pregnancy, menopause, physical disability, neuroendocrine conditions, and the gut microbiome. Environmental factors concern culture, norms, ethnicity, environmental and chemical pollution, socioeconomic factors, and obesogenic environment. Behavioral factors include overeating, excessive consumption of high-calorie foods, sedentary lifestyle, physical inactivity, diet, quitting smoking, and insufficient sleep [2, 10, 12].

Because many risk factors are associated to obesity, it is essential to have a thorough understanding of the general knowledge and behavior of the population [13]. So, studies that aim to assess and analyze knowledge, attitude, and practices (KAP) are more suitable [14]. Moreover, KAP studies are essential when it comes to evaluate interventions related to communication and nutrition education, namely, activities explicitly aimed at improving the KAP of populations in terms of nutrition [15]. However, the only study that has been carried out in Cameroon regarding KAP on obesity and overweight reveals a low prevalence of obesity with gender distribution and a satisfactory attitude towards physical activity. However, attitudes related to eating disorders, antenatal influence, and intrinsic factors of modern civilization were found to be not satisfactory, which may put the study population at risk for obesity. Moreover, this work was done on small group which may not be representative of the entire population. Thus, to contribute to the fight against obesity in Cameroon, the objective of this study was to collect basic indicators on the KAP of the populations concerning overweight and obesity in Douala City and Manjo (urban and rural area, respectively) in Cameroon. This will allow to identify the factors that influence the occurrence and management of obesity and therefore to formulate the relevant recommendations to guide the interventions.

## 2. Material and Methods

### 2.1. Study Area

The present study was carried out in two localities of Littoral Region-Cameroon, precisely Douala City and Manjo downtown. Douala (altitude: 19 m; latitude: 4°2′53^″^N; longitude: 9°42′15^″^E; 210 km^2^) is the economic capital of Cameroon and the main business center in Central Africa. It is located on both banks of the Wouri River and is organized into 06 subdivisions. The population is estimated at 4 million inhabitants in 2015 with a growth rate of 6.4%. Although the indigenous peoples are the Bassas, the Douala, and the Bakokos, Douala is today a mosaic of different Cameroonian ethnic groups. Manjo (altitude: 526 m; latitude: 4°50′34^″^N; longitude: 9°49′18^″^E; 305 km^2^) is a municipality of Moungo Division, located in the northern part of the Littoral region. It is spread over two cantons and 33 villages and has a population of 37,661 inhabitants in 2015. Manjo downtown is organized in 9 districts. The indigenous people is Mbo'o, and the main foreign ethnic groups are Bamiléké, Hausa, Bakaka, Bamenda, Bassa, Diboum, Ewondo, Bororo, and Yabassi. The main economic activity in the commune of Manjo is agriculture. Besides, as the other municipalities of Moungo, it is a large basin of production of fruits and vegetables in Cameroon supplying several zones of the country including Douala [16].

### 2.2. Study Population and Setting

This study was an epidemiological community-based cross-sectional descriptive study conducted from March to June 2022. The target population for this study included individuals of both sexes who were at least 18 years of age or older resident for at least 6 months in Douala or Manjo. Persons suffering from disability and movement disorders and psychiatric disorders (anorexia, bulimia, depression, and bipolar disorders) were excluded from the study. The sampling method in this study was nonprobability sampling involving nonrandom selection based on convenience. In Douala, campaigns for the recruitment of participants were organized in the main public places including markets, major crossroads, hospitals, and sports halls/places. Likewise, in Manjo downtown, the recruitment was done in the main markets, hospitals, and chiefdoms. The recruitment was done with the agreement of local authorities.

Calculation of sample size was done to ensure the minimum number of respondents needed to be a representative sample for the whole studied populations. The sample size was determined using the following formula: *n* = *z*^2^*pq*/*i*^2^, where *n* is the sample size, *z* is the *Z* statistic for a level of confidence (1.96 for 95% CI), *p* is the estimative prevalence (unknown in this study, we used 50%), and *i* is the margin of errors (0.05). Thus, the calculated sample size for each studied area was 385. Finally, a total of 897 questionnaires were filled out at Douala and 341 at Manjo.

This study was approved by University of Douala Institutional Ethics Committee (Ethical Clearance No. 2714 CEI-Udo/06/2021/M). The informed consent was obtained from each participant.

### 2.3. Questionnaire Design and Data Collection

Data collection was conducted by trained instructors. Seven teams of two persons each were constituted, five for Douala and two for Manjo. A multisection questionnaire of four parts was used in this research. The first section was focused on demographic and medical characteristics, and the third others were focused on KAP information, especially knowledge, attitude, and practices. In Manjo, data were collected with the assistance of associations of young peoples who facilitated the exchanges between population and our team by translating the information from French language to local language and vice-versa so that the illiterates can understand.

The sociodemographic data of the participants were firstly collected in particular, age, gender, origin region, religion, occupation, education, lifestyle, and marital status. Information on the KAP consisted of 29 questions including 08 for knowledge, 11 for attitudes, and 10 for practices. The questionnaire was designed based on the model proposed by the FAO for conducting KAP surveys on obesity [14] and a study conducted in China by Xue et al. [2].

Then, their weight and height were measured, and their body mass index (BMI) was calculated. The height was measured in a standing position and without shoes, using a standard strip meter with a precision of 0.5 cm, and the weight was measured using a dial scale with a precision of 0.5 kg. BMI was evaluated using the formula: BMI (kg/m^2^) = weight/(height)^2^. It was used to determine statutes of respondents according to WHO references: underweight <18.5 kg/m^2^, normal 18.50–24.99 kg/m^2^, overweight ≥25 kg/m^2^, and obese ≥30 kg/m^2^.

The questionnaire was checked for validity and consistency in our study population. Before starting this study, a pilot test was conducted with 30 individuals from each area. Minor modifications (e.g., rephrasing) were made based on this pilot study.

### 2.4. Data Handling and Statistical Analysis

Data were entered into a MS Excel spreadsheet and organized for statistical analysis. Data were treated according to the study areas and the respondents' BMI. Frequency and relative frequency were calculated using SPSS 20 (IBM Corp.). For the determination of the impact of sociodemographic parameters on the KAP, a score varying from 0 to 100 was attributed to each question. Less than 50 considered as poor, 50 to 75 as moderate, and 75 to 100 considered as good [17]. For each respondent, a mean score was calculated to define knowledge, attitude, and practice variables. For a given parameter, the mean and standard deviation of each variable were evaluated. Finally, correlations between knowledge, attitude, and practice were determined. Inferential statistics tests used were *χ*^2^ test, independent Student *t*-test, ANOVA (followed by Tukey's post hoc test), and Pearson correlation coefficient. The values of *P* < 0.05 were considered significant.

## 3. Results and Discussion

Overall, of the 1,238 completed questionnaires, 26 were ignored due to incomplete or incoherent information, and the result analysis was conducted on 1,212 questionnaires among which 892 (73.6%) from Douala and 320 from Manjo (26.4%).

### 3.1. Sociodemographic Characteristics and BMI of Participants

#### 3.1.1. Sociodemographic Characteristics and BMI of Participants according to Study Site

Table 1 presents the socio-demographic parameters and the BMI of the respondents according to the study site. The age group over 50 years is poorly represented in the study population, with only 3% in Douala and 9.1% in Manjo. On the other hand, the ≤30 years (71.4%) are the most represented in Douala. Respondents with a university background had more encounters in Douala (68%). The respondents from Manjo are recruited in all activity sectors, while in Douala students are highly represented. The largest proportion of Douala respondents is single (79%), while in Manjo most of them are married (65%). The prevalence of overweight (34.1%) and obesity (50.0%) are higher in Manjo as compared to Douala.

The fact that most of the respondents are ≤30 years old in Douala and between 30 and 50 years old in Manjo could be explained by the fact that Douala is a student city and the probability of meeting young people during the surveys was very high, compared to Manjo which is an agricultural town where the main activity is farming. In Cameroon, young people generally abandon agriculture which could justify why they are rarely met in rural areas [18]. The higher diversity of universities in Douala could also explain why we found many students there and why more respondents have a university background. Moreover, it is not surprising to have more single respondents in Douala. Indeed, according to BUCREP, the average age at first marriage for men in Cameroon is estimated at 21.41-27.12 years in 2005 and this age range is much higher for those studying at university levels. The prevalence of overweight and obesity is much higher than those found by Nansseu, Noubiap, and Bigna [19] in Cameroon in general (respectively, 26.0% and 15.1%). This difference in prevalence estimates could be explained by differences in the methodology and data used since the previous authors have used a systematic review and meta-analysis of overweight and obesity among adults, while the current study is an epidemiological community-based cross-sectional descriptive study conducted in two localities only. Our estimates were based only on field data, while Nansseu, Noubiap, and Bigna [19] derived their estimates from empirical data. However, our prevalence of overweight at 30.0% and 34.1%, respectively, in Douala and Manjo are higher than the estimates of the central sub-Saharan African region (24.8%-25.7%) [20]. This result may suggest that our sampling was carried out in a cluster of overweight and obesity or that the prevalence is on rise in Cameroon.

The high proportion of overweight and obesity in Manjo is not surprising. Indeed, Simo et al. [7] have also found a higher prevalence of obesity and overweight in rural areas than in urban. The abundance and diversity of low-cost agricultural products in rural areas could facilitate access to food for people living there, which could lead to an increase in the consumption of high-calorie foods. Additionally, the study found that respondents in Manjo are generally more active and have higher purchasing power, which could allow them to afford any type of food compared to those in Douala who may be more financially dependent.

#### 3.1.2. Sociodemographic Characteristics of Participants according to BMI

Table 2 presents the distribution of respondents' sociodemographic characteristics according to the BMI. The different categories observed in this study were normal weight, overweight, and obesity. Results reveal that age significantly influences the occurrence of overweight and obesity. Indeed, the ≤30 years are more affected by being overweight (57.9%), while the age range [30-50] is more affected by obesity (57.3%). Students suffer the most from being overweight (49.8%), while for obesity there was a homogeneous distribution of respondents according to occupations. Single people are more likely to be overweight (64.0%), yet married people are significantly affected by obesity (62.3%). Gender, region of origin, religion, and level of education do not significantly influence the occurrence of overweight and obesity.

Al-Ghamdi et al. [21] have already observed an increase in the prevalence of obesity with age. These authors have observed that a high age could constitute a risk factor for obesity. This could be due to a number of factors, including a decrease in physical activity levels, changes in hormonal balance, and changes in dietary habits. It is also possible that the consequences of poor hygiene and dietary practices become more apparent with age, due to the weakening of the immune system. As individuals age, their ability to fight off infections and illnesses may decrease, making them more susceptible to the negative effects of an unhealthy diet and lifestyle. Moreover, it is not surprising that the highest prevalence of overweight respondents is found among students and therefore those ≤30 years old. Current literature indicates that young adults are at risk of becoming obese or gaining excess weight when transitioning from childhood into adolescence or from adolescence into adulthood [22]. The high prevalence of obesity in married respondents in this study is not an epi-phenomenon. In China, Liu et al. [23] even demonstrated that marriage is a risk factor for obesity. However, in Africa, very few studies have established a correlation between marriage and obesity. Nevertheless, it is hypothesized that marriage creates permanent stress due to the social pressures it generates, which promotes weight gain. Indeed, according to Meltzer et al. [24], marital strain causes stress that interferes with self-regulatory behaviors, thus favor weight gain.

### 3.2. Knowledge of Obesity among the Population of Douala and Manjo

#### 3.2.1. Knowledge according to the Study Site

Table 3 presents respondents' knowledge on overweight and obesity according to the study site. The study site does not influence whether you have ever heard of obesity, the knowledge of the definition of obesity, and the knowledge related to the prevention means. Living in Manjo increases the consideration that obesity is a disease (*P* = 0.0253). Moreover, the proportion of respondents who think that obesity is hereditary is significantly higher in Manjo (65.9%) than in Douala (52.0%). Recognition of the BMI as a diagnostic tool for overweight and obesity is greater in Manjo. Manjo's respondents have good knowledge on the consequences of obesity on health and its major causes (*P* < 0.0001). At this level, rural area has a positive effect on general knowledge about obesity. These results are quiet surprising since the level of education of the respondents in Douala is higher.

#### 3.2.2. Knowledge according to BMI

The data on the effect of BMI on knowledge related to overweight and obesity are recorded in Table 4. The BMI has no influence on the fact of having already heard of obesity, on whether obesity is a disease, on its definition, on the knowledge of the BMI as an indicator of obesity, and on the means of prevention. However, being obese (59.0%) or overweight (61.6%) significantly improves the belief that obesity is hereditary (*P* = 0.0126) and the knowledge of the associated causes (*P* = 0.0027) and consequences (*P* = 0.0234). These results suggest that obese and overweight respondents are only aware of the health risk that their situation could induce. However, they do not know much on the means of prevention.

### 3.3. Attitude of Obesity among the Population of Douala and Manjo

#### 3.3.1. Attitude to Study Site

The attitude regarding obesity among the population of Douala and Manjo is depicted in Table 5. The proportion of respondents (31%) who describe themselves as compulsive eaters is significantly higher in Manjo as compared to Douala (23.0%). The same trend is observed for respondents who pay less attention to their bodies (28.1% in Manjo and 12.0% in Douala). Regarding the confidence to practice physical exercises, there is a significant difference between the two areas (only 38.2% in Manjo and 61.0% in Douala). The proportion of respondents who do not care about their weight was significantly high in Manjo (26.9%). In addition, the lowest proportion of respondents (43.1%) who believe that weight management is an important strategy to maintain good health was found in Manjo. However, there is a significant difference between the respondents of the two localities in terms of eating smaller portions of food to control their weight. This trend was more important in Manjo (55%) against only 47% in Douala. Living in Douala significantly improves the perception of respondents on their weight. The attitudes regarding obesity are poor in Manjo although the fact that this area presents the higher knowledge.

#### 3.3.2. Attitude according to BMI

The distribution of the respondents' attitudes according to the BMI is recorded in Table 6. Being normal weight, overweight, or obese is not significantly associated with being a compulsive eater. The same is true for the perception of obesity, the practice of physical activity, and difficulties in practicing physical activity. On the other hand, having a normal weight significantly increases the attention paid by respondents to their bodies (40.5%). Being overweight and obese significantly alters respondents' perceptions of their bodies. Indeed, up to 73.6% of overweight people and 66.6% of obese people think they are rather skinny, thin, or normal weight. Moreover, a large proportion of them thinks it is very difficult for them to become obese (53.1% and 42.4% for obese and overweight, respectively). Being overweight and obese significantly increases the proportion of respondents who believe that eating little can help fight obesity. However, most of them admit that it is difficult to reduce their food intake the (22.9% for overweight respondents and 34.5% for obese). These results suggest that obese or overweight respondents have a poor perception of their appearance. This may be what hinders their efforts to respect hygiene and dietetic rules.

### 3.4. Practices Related to Obesity among the Population of Douala and Manjo

#### 3.4.1. Practices according to the Study Site

Table 7 presents the practices related to obesity among Douala and Manjo respondents. The respondent's place of residence does not influence breakfast consumption. The same is true for the criteria for choosing food and the type of entertainment practiced by the respondents. Dessert intake is significantly higher in Manjo than in Douala (*P* < 0.0001). In addition, fast food consumption is significantly higher in Manjo compared to Douala (*P* = 0.0003). Similar trends were observed for the consumption of milk and dairy products, fruits/vegetables, herbal teas and hot drinks, and big meals. Befort et al. [25] have also documented a high consumption of fast food in rural area, suggesting the effect of food transition.

#### 3.4.2. Practices according to BMI

Table 8 presents the practices related to obesity according to BMI. The proportion of obese people who consume more than two desserts per day is significantly higher than that of normal weight (15.5%). Being obese or overweight significantly favors the consumption of fast food (*P* = 0.0045). The same is true for the consumption of hot drinks such as tea and herbal teas (*P* = 0.0191). On the other hand, obesity or overweight leads to a significant drop in the practice of physical activity (13.2% and 14.1%, respectively, in overweight and obese people against 28.0% in normal-weight people). However, BMI does not influence practices such as eating breakfast, consumption of milk/yoghurt or soymilk, consumption of fruits/vegetables, modification of food size in bad mood periods, the choice of food based on its nutritional value, and the type of entertainment chosen to spare time. Overall, the study suggests that there are differences in certain practices related to obesity.

### 3.5. Knowledge, Attitude, and Practices Scores according to the Study Site, BMI, and Sociodemographic Characteristics of Participants

Scores have been defined in order to determine the impact of different parameters on the KAP.  Table 9 presents these scores according to the study site, BMI, and sociodemographic characteristics. Concerning the knowledge score, it varies from poor (less than 50) to moderate (50-75) according to the classification of Baig et al. [17]. We can therefore conclude that the knowledge of the respondents is low. More specifically, the respondents from Manjo (66.7 ± 20.4) have the highest score as compared to those of Douala. Obese respondents (59.7 ± 17.9) also have the highest knowledge score. This score is lower among respondents with primary studies background or none (42.2 ± 23.6). This trend is confirmed by the fact that respondents whose occupation is students have the highest score (54.8 ± 27.2). Respondents living with a family (54.0 ± 28.5) have a higher knowledge score than those who live alone or other (48.0 ± 29.3). This trend is confirmed by the fact that married respondents have a better score than single/divorced/widow respondents. Muslim respondents have the lowest knowledge score (46.2 ± 26.5).

Regarding the attitude score, it varies from poor to moderate suggesting that actions should be taken to improve the perception of respondents against obesity and overweight. However, respondents from Douala (52.12 ± 17.3) have the best score compared to those from Manjo (46.1 ± 19.2). Being obese also increases the attitude score (67.3 ± 17.8). Women have the best score as compared to men (68.1 ± 18.5). In addition, being less or equal to 30 years old (68.3 ± 20.2) or more than 50 years old (66.7 ± 17.8) improves attitude towards obesity and being overweight. Having studied at university improves the attitude score (68.7 ± 20.1) compared to the other levels of study. Respondents who have the best attitude score are the employee (67.1 ± 18.1) and the self-employee (68.8 ± 20.3).

In general, the practice score is poor (less than 50) and this can justify the high prevalence of overweight and obesity in our study population. Otherwise, the practice score is higher in Manjo (52.1 ± 17.3) as compared to Douala (46.1 ± 19.2). Contrary to the attitudes score where women had the highest value, we found that men have the best practices score (48.9 ± 16.8). We also note that respondents with more than 50 years have the best practice score (56.62 ± 17.8). Respondents with the highest levels of education have the highest practice scores, while the unemployed ones have the lowest score. Married respondents have the best score (50.1 ± 17.6) than the single/divorced/widow. When it comes to religion, Christian and Islamic respondents have the lowest practice score.

Knowledge about overweight and obesity is surprisingly greater in Manjo than in Douala, yet we expected the opposite trend. This knowledge ranges from the definition of obesity, strategic diagnostic tools, and even the consequences. These observations are surprising on several counts, namely, a large proportion of respondents in Douala have a university level of study and a high level of knowledge of the populations of Manjo. In general, several awareness initiatives on health issues in Cameroon are directed towards rural areas to fulfil the infrastructural gap that characterizes them. Unlike other KAP studies, which generally focus on the risk factors of obesity, this study has shown that being obese improves the knowledge score on this pathology. This result shows that obese or overweight people generally question their condition and end up being better informed on the subject. Having at least secondary education improves the knowledge score. This is even truer as having a student occupation improves this score. Abdulrahman and Alnagar [26] made similar observations. They have demonstrated that university students had significantly higher knowledge of risk factors for obesity than primary and middle school students in Saudi Arabia. Lifestyle positively influences the knowledge score. Indeed, living with a family or being married increases this indicator. However, several studies have shown that living with a husband or family could constitute a risk factor for obesity [27]. This observation suggests that the presence of the family serves as a channel for amplifying knowledge about obesity even though they are not always applied. Belonging to the Muslim religion lowers the knowledge score of respondents. Indeed, in Cameroon, the literacy rate in the Muslim community is generally low [28]. However, in this study, we observed that a high level of study improved the knowledge score.

In Cameroon, only one study was able to establish the attitude of a group of university students towards obesity [15]. This study reveals a satisfactory attitude related to the sedentary of the study population. Thus, those students willingly practice physical activities. We also obtained the best attitude score with respondents who had reached university level. However, Mengue and Enyegue [15] did not provide information on other factors influencing attitude. In this study, we were able to associate this indicator with parameters such as study site, BMI, gender, age, and occupation.

In addition, the practice score varies according to factors such as study site, gender, age, education level, occupation, marital status, and religion. This result reveals that many socioeconomic factors influence the practices related to obesity. In Cameroon, as in several African countries, obesity is more prevalent in rural areas than in urban areas [19]. However, this study reveals best practices relating to overweight and obesity precisely in rural areas. This implies that other determiners not elucidated by this study must be considered. Concerning gender, the results obtained could justify the fact that women are the most affected by obesity in Cameroon [19] because they have the least good practices. Level of education showed a significant effect on KAP scores which also supports several studies [29]. We found that respondents with a university or a secondary studies background had higher practice scores than the primary one. In this study, the mean practice score was higher in those who have employment and students. Another study conducted in Pakistan also showed that the mean practice score of housewives and blue-collar workers was lower than that of white-collar workers [29]. It may be due to the accessibility of the information or lack of awareness. The married presents the best practices, yet the work of Liu et al. [23] demonstrated that marriage is a risk factor for obesity. This demonstrates that other aspects of marriage such as food intake could promote the occurrence of overweight and obesity. Regarding the effect of religion on the practice score, Bharmal et al. [30] have demonstrated that respondents affiliated with Hinduism, Sikhism, and Islam compared to those with no religious affiliation have a high risk of being overweight/obese. Our study suggests that Christianity and Islam reduce the practice score since those from other religions exhibited the highest score.

### 3.6. Correlation between Knowledge, Attitude, and Practice Scores

Table 10 presents the correlations between knowledge, attitude, and practices. There is no significant correlation between knowledge and attitude scores (*r* = 0.195). This suggests that there is no association between the knowledge of the respondents and their attitudes towards obesity. The results also show that the attitude score is not significantly correlated to the practice score (*r* = 0.253). Moreover, we found a positive correlation between knowledge score and practice score (*r* = 0.578). These results are not in agreement with those of Saleh et al. and Mohd Hatta et al. [29, 31] who showed that knowledge affects attitude and attitude affects practice. However, according to Essi and Oudou [32], attitudes are the gap between knowledge and practices and result from the various constraints weighing on the person. From this point of view, the choice of variables representing attitudes is tricky if we want to be able to establish the gap in knowledge and practices. The differences can be explained by the choice of variables defining attitude. In this study, we choose determinants such as anxiety, self-esteem, and self-confidence. However, for Saleh et al. [29], attitude statements were related to knowledge.

## 4. Conclusion

This study identified the factors that were associated with KAP and determined the correlation between KAP, BMI, and place of residence of adults in Douala and Manjo. This study reveals a poor or moderate knowledge and attitude with poor practice among the overall population. Moreover, the study revealed a positive correlation between knowledge and practice scores, suggesting that once knowledge is acquired, practice follows. However, we also observed that residing in a rural area (Manjo), being overweight or obese, having completed secondary education, and being married are the parameters that improve the knowledge and the practice scores. Consequently, to fight against obesity, attitudes of respondents need to be changed. Indeed, the awareness of student on lifestyle and dietary rules could constitute an approach to reduce the disarticulation between knowledge and attitude on the one hand and attitude and practices on the other hand. Besides, we were able to identify areas of intervention based on particular on the management of parameters such as anxiety, self-esteem, and self-confidence. However, a well-designed prospective study with a large sample size is recommended in order to get accurate results so that it can be generalized to the population.

## Figures and Tables

**Table 1:** Sociodemographic characteristics and BMI of participants according to study site.

Parameters	Frequency (%)		*P*
	Douala (*n* = 892)	Manjo (*n* = 320)	
Age (years)			
≤30	71.4%	36.9%	0.0001
[30-50]	25.6%	54.0%	
>50	3.0%	9.1%	
Gender			
Men	40.0%	46.9%	ns
Women	60.0%	53.1%	
Origin region			
Adamawa/north/far north	5.0%	6.8%	ns
Centre/east/south	23.0%	19.1%	
Littoral/southwest	22.0%	34.4%	
West/northwest	50.0%	39.7%	
Religion			
Christian	89.0%	89.1%	ns
Muslim	8.0%	5.9%	
Other	3.0%	5.0%	
Occupation			
Student	63.0%	30.0%	<0.0001
Employee	9.0%	20.0%	
Self-employment	19.0%	20.0%	
Unemployed	9.0%	30.0%	
Education			
Primary studies	7.0%	7.8%	0,0012
Secondary studies	24.0%	52.8%	
University studies	68.0%	37.8%	
None	1.0%	1.6%	
Lifestyle			
Life alone	18.0%	10.9%	ns
Life with family	81.1%	85.9%	
Other	1.0%	3.2%	
Marital status			
Single	79.0%	30.9%	<0.0001
Married	20.0%	65.0%	
Divorced	0.0%	0.9%	
Widow	1.0%	3.2%	
BMI			
Low weight (BMI < 18.5)	0.0%	0.0%	<0.0001
Normal weight (BMI [18.5–25])	54.9%	15.9%	
Overweight (BMI [25–30])	30.0%	34.1%	
Obesity (BMI ≥ 30)	14.1%	50.0%	

ns: not significant. *P* < 0.05.

**Table 2:** Sociodemographic characteristics of participants according to BMI.

Parameters	Frequency (%)			*P*
	Normal BMI [18.5–25] (*n* = 547)	Overweight BMI [25–30] (*n* = 375)	Obese BMI ≤ 30 (*n* = 290)	
Age (years)				
≤30	33.6%	57.9%	30.7%	0.0003
[30-50]	17.5%	37.0%	57.3%	
>50	1.8%	5.1%	12.0%	
Gender				
Men	44.3%	41.1%	39.0%	ns
Women	55.7%	58.9%	61.0%	
Origin region				
Adamawa/north/far north	6.6%	5.6%	3.5%	ns
Centre/east/south	24.6%	23.5%	16.6%	
Littoral/southwest	23.2%	29.3%	22.7%	
West/northwest	45.6%	41.6%	57.2%	
Religion				
Christian	87.9%	90.9%	87.2%	ns
Muslim	8.1%	6.9%	5.5%	
Other	4.0%	2.2%	7.3%	
Occupation				
Student	73.6%	49.8%	25.6%	<0.0001
Employee	5.6%	13.1%	21.3%	
Self-employment	12.2%	21.1%	29.0%	
Unemployed	8.6%	16.0%	24.1%	
Education				
Primary studies	4.4%	6.9%	11.7%	ns
Secondary studies	22.6%	32.0%	48.2%	
University studies	73.0%	58.9%	39.0%	
None	0.0%	2.2%	1.1%	
Lifestyle				
Life alone	17.7%	20.0%	8.0%	ns
Life with family	80.5%	78.9%	90.7%	
Other	1.8%	1.1%	1.3%	
Marital status				
Single	85.7%	64.0%	32.8%	<0.0001
Married	14.3%	33.8%	62.3%	
Divorced	0.0%	1.1%	1.1%	
Widow	0.0%	1.1%	3.8%	

ns: not significant. *P* < 0.05.

**Table 3:** Knowledge of obesity among the population of Douala and Manjo according to the study site.

	Frequency (%)		*P*
	Douala (*n* = 892)	Manjo (*n* = 320)	
Have you ever heard of obesity?			
Yes	97.0%	95.0%	ns
No	3.0%	5.0%	
Do you think obesity is a disease?			
Yes	76.0%	88.1%	0.0253
No	24.0%	11.9%	
Do you think that obesity is hereditary?			
Yes	52.0%	65.9%	0.0153
No	48.0%	34.1%	
How do you define obesity?			
Self-perception	54.0%	21.9%	ns
By scientific calculation	35.0%	35.9%	
Other evaluation	25.0%	11.9%	
Do you know the BMI prescribed by the World Health Organization?			
Very well	29.0%	40.9%	0.0058
Somewhat familiar	34.0%	40.9%	
Not at all familiar	37.0%	18.2%	
What are the health problems that can occur when a person is overweight or obese?			
Increased risk of chronic conditions (such as heart/cardiovascular disease, high blood pressure, diabetes, stroke, certain types of cancer, respiratory difficulties, chronic musculoskeletal problems, skin problems, and infertility)	84.0%	91.9%	<0.0001
Reduced quality of life	28.0%	75.0%	
Premature death	26.0%	63.1%	
Other	6.1%	43.1%	
Do not know	5.0%	4.1%	
Can you tell me the reasons why people are overweight or obese?			
Increased/excessive intake of energy-dense foods that are high in fat and/or sugar	84.0%	93.1%	<0.0001
Lack of or decreased physical activity	57.0%	85.0%	
Sedentary lifestyle	21.0%	70.0%	
Heredity	28.0%	69.1%	
Psychological factors	20.0%	51.9%	
Medication	15.0%	40.9%	
Other	4.0%	26.9%	
Do not know	2.0%	1.9%	
How can people prevent overweight and obesity?			
Reduce energy intake (less high-energy foods and drinks)/reduce the intake of fatty and sugary foods	74.0%	91.9%	ns
Eat vegetables and fruits more often	51.0%	81.9%	
Eat legumes/whole-grain products more often	20.0%	68.1%	
Regularly consume hot drinks (teas, tisanes…)	40.0%	66.9%	
Increase physical activity level/engage in regular physical activity	60.0%	58.1%	
Other	5.0%	26.9%	
Do not know	1.0%	0.9%	

ns: not significant. *P* < 0.05.

**Table 4:** Knowledge of obesity among population of Douala and Manjo according to BMI.

	Frequency (%)			*P*
	Normal weight BMI [18.5-25] (*n* = 547)	Overweight BMI [25-30] (*n* = 375)	Obesity BMI ≤ 30 (*n* = 290)	
Have you ever heard of obesity?				
Yes	96.7%	97.6%	92.8%	ns
No	3.3%	2.4%	7.2%	
Do you think obesity is a disease?				
Yes	80.8%	76.5%	78.6%	ns
No	19.2%	23.5%	21.4%	
Do you think that obesity is hereditary?				
Yes	48.4%	61.6%	59.0%	0.0126
No	51.6%	38.4%	41.0%	
How do you define obesity?				
Self-perception	42.4%	48.3%	50.0%	ns
By scientific calculation	33.3%	30.7%	43.8%	
Other evaluation	26.0%	23.2%	13.1%	
Do you know the BMI prescribed by the World Health Organization?				
Very well	33.3%	30.4%	31.7%	ns
Somewhat familiar	33.5%	38.4%	37.9%	
Not at all familiar	33.2%	31.2%	30.4%	
What are the health problems that can occur when a person is overweight or obese?				
Increased risk of chronic conditions (such as heart/cardiovascular disease, high blood pressure, diabetes, stroke, certain types of cancer, respiratory difficulties, chronic musculoskeletal problems, skin problems, and infertility)	86.3%	84.5%	87.2%	0.0234
Reduced quality of life	29.3%	40.8%	59.3%	
Premature death	27.6%	35.2%	52.8%	
Other	9.3%	16.3%	28.3%	
Do not know	4.4%	4.3%	7.2%	
Can you tell me the reasons why people are overweight or obese?				
Increased/excessive intake of energy-dense foods that are high in fat and/or sugar	85.9%	86.1%	85.9%	0.0027
Lack of or decreased physical activity	59.0%	61.6%	76.6%	
Sedentary lifestyle	21.9%	36.3%	53.8%	
Heredity	31.3%	39.7%	52.8%	
Psychological factors	19.7%	29.6%	41.4%	
Medication	16.1%	21.6%	33.8%	
Other	4.9%	6.7%	23.8%	
Do not know	1.3%	1.3%	4.1%	
How can people prevent overweight and obesity?				
Reduce energy intake (less high-energy foods and drinks)/reduce the intake of fatty and sugary foods	75.7%	77.9%	86.6%	ns
Eat vegetables and fruits more often	52.8%	57.9%	72.1%	
Eat legumes/whole-grain products more often	21.8%	31.2%	44.1%	
Regularly consume hot drinks (teas, tisanes…)	40.0%	45.9%	61.7%	
Increase physical activity level/engage in regular physical activity	60.1%	54.4%	66.9%	
Other	6.8%	7.5%	23.4%	
Do not know	0.7%	1.3%	1.0%	

ns: not significant. *P* < 0.05.

**Table 5:** Attitude of obesity among population of Douala and Manjo according to study site.

	Frequency (%)		*P*
	Douala (*n* = 892)	Manjo (*n* = 320)	
Are you a picky eater?			
Yes	23.0%	31.9%	0.032
No	77.0%	68.1%	
Do you care about your body shape?			
Not at all	12.0%	28.1%	0.0003
Somewhat	53.0%	55.0%	
Very much	35.0%	16.9%	
What body shape do you think you have?			
Skinny	9.0%	4.1%	0.0010
A little thin	14.0%	10.9%	
Normal	55.0%	38.1%	
A little fat	20.0%	40.0%	
Very fat	2.0%	6.9%	
What is your attitude towards your weight?			
I do not care. I just want to be comfortable	11.0%	26.9%	0.0051
Weight is important and needs to be maintained for good health	58.0%	43.1%	
On the premise of ensuring good health, you should lose weight for a good figure	36.0%	43.1%	
In order to have a good figure, you should sacrifice your health a little	6.1%	33.1%	
I have no time to consider weight and health problems because I am busy studying	2.0%	14.1%	
I recognize obesity is a problem, but losing weight is too hard	16.0%	33.1%	
How likely do you think you are to become obese?			
Likely	26.0%	26.9%	ns
You are not sure	35.0%	30.0%	
Not likely	39.0%	43.1%	
How serious do you think it is to be obese?			
Not really serious	13.0%	15.9%	ns
Neutral/serious	16.0%	20.0%	
Serious	71.0%	64.1%	
How good do you think it is to eat less, for example by eating smaller portions of food?			
Not good	35.0%	18.2%	0.0321
You are not sure	18.0%	25.9%	
Good	47.0%	55.9%	
How difficult is it for you to eat less?			
Not difficult	33.0%	39.0%	ns
So-so	47.0%	38.0%	
Difficult	20.0%	23.0%	
How good do you think it is to do some physical activity, such as walking for 30 minutes a day, running, or doing a sport?			
Not good	2.0%	7.8%	0.0020
You are not sure	11.0%	23.1%	
Good	87.0%	69.1%	
How confident do you feel in doing some physical activity/exercise?			
Not confident	10.0%	10.9%	0.0122
Ok/so-so	29.0%	50.9%	
Confident	61.0%	38.2%	

ns: not significant. *P* < 0.05.

**Table 6:** Attitude of obesity among population of Douala and Manjo according to BMI.

	Frequency (%)			*P*
	Normal weight BMI [18.5-25] (*n* = 547)	Overweight BMI [25-30] (*n* = 375)	Obesity BMI ≤ 30 (*n* = 290)	
Are you a picky eater?				
Yes	22.9%	28.5%	28.6%	ns
No	77.1%	71.5%	71.4%	
Do you care about your body shape?				
Not at all	13.2%	18.1%	18.6%	0.0071
Somewhat	46.3%	61.6%	58.6%	
Very much	40.5%	20.3%	22.8%	
What body shape do you think you have?				
Skinny	12.8%	5.1%	2.8%	<0.0001
A little thin	18.8%	12.8%	2.4%	
Normal	60.5%	55.7%	61.4%	
A little fat	7.9%	25.1%	22.8%	
Very fat	0%	1.3%	10.6%	
What is your attitude towards your weight?				
I do not care. I just want to be comfortable	12.4%	19.7%	13.8%	<0.0001
Weight is important and needs to be maintained for good health	63.3%	48.3%	43.8%	
On the premise of ensuring good health, you should lose weight for a good figure	31.3%	39.7%	48.6%	
In order to have a good figure, you should sacrifice your health a little	6.9%	13.3%	24.1%	
I have no time to consider weight and health problems because I am busy studying	2.6%	4.3%	10.7%	
I recognize obesity is a problem, but losing weight is too hard	10.1%	20.5%	37.9%	
How likely do you think you are to become obese?				
Likely	32.2%	25.9%	14.5%	0.0023
You are not sure	36.4%	31.7%	32.4%	
Not likely	31.4%	42.4%	53.1%	
How serious do you think it is to be obese?				
Not really serious	13.5%	17.1%	11.0%	ns
Neutral/serious	16.1%	14.9%	21.4%	
Serious	70.4%	68.0%	67.6%	
How good do you think it is to eat less, for example by eating smaller portions of food?				
Not good	40.8%	28.5%	11.7%	<0.0001
You are not sure	20.3%	17.9%	22.1%	
Good	38.9%	53.6%	66.2%	
How good do you think it is to do some physical activity, such as walking for 30 minutes a day, running, or doing a sport?				
Not good	2.9%	4.8%	0.7%	ns
You are not sure	10.4%	14.7%	20.0%	
Good	86.7%	80.5%	79.3%	
How difficult is it for you to eat less?				
Not difficult	45.3%	36.0%	22.4%	0.0012
So-so	39.9%	41.1%	43.1%	
Difficult	14.8%	22.9%	34.5%	
How confident do you feel in doing some physical activity/exercise?				
Not confident	9.0%	9.9%	12.1%	ns
Ok/so-so	28.5%	39.7%	41.0%	
Confident	62.5%	50.4%	46.9%	

ns: not significant. *P* < 0.05.

**Table 7:** Practices related to obesity among the population of Douala and Manjo according to study site.

	Frequency (%)		*P*
	Douala (*n* = 892)	Manjo (*n* = 320)	
How often do you eat breakfast each week?			
<2 days	30.0%	16.8%	ns
2-3 days	32.0%	40.0%	
3-5 days	24.0%	24.1%	
>5 days	14.0%	19.1%	
How many times have you had desserts in the past 7 days?			
None	32.0%	10.9%	<0.0001
1 time	23.0%	12.2%	
2-6 times	24.0%	31.9%	
Once every day	16.0%	30.0%	
Twice a day or more	5.0%	15.0%	
In the past 7 days, how many days did you eat fast food?			
None	47.9%	15.0%	0.0003
1 day	19.1%	30.0%	
2 days	17.0%	30.9%	
3 days	9.0%	15.9%	
4 days and more	7.0%	8.2%	
In the past 7 days, how many days did you have at least one glass of milk/yogurt or soymilk?			
None	34.1%	11.9%	0.0002
1 day	22.9%	16.9%	
2 days	21.0%	33.4%	
3 days	13.0%	25.9%	
4 days and more	9.0%	11.9%	
In the past 7 days, how many days did you eaten fruits/vegetables?			
None	13.0%	6.9%	0.0064
1 day	18.0%	9.4%	
2 days	27.0%	20.9%	
3 days	22.0%	35.9%	
4 days and more	20.0%	26.9%	
In the past 7 days, how many days did you taken hot drinks (tea, tisanes...)?			
None	40.0%	10.0%	<0.0001
1 day	13.0%	10.9%	
2 days	18.0%	14.1%	
3 days	13.0%	21.9%	
4 days and more	16.0%	43.1%	
When you are in a bad mood, do you choose to eat a big meal?			
Yes	16.0%	30.0%	0.0187
No	84.0%	70.0%	
Do you choose foods based on their nutritional value in your daily meals?			
Always	13.0%	10.9%	ns
Often	41.0%	48.1%	
Rarely	29.0%	34.1%	
Not at all	17.0%	6.9%	
How many times do you do physical activity/exercise per month?			
None	20.0%	20.0%	0.0057
1 time	13.0%	23.1%	
2 times	14.0%	20.0%	
3 times	11.0%	11.9%	
4 times	11.0%	14.1%	
5 times	7.0%	4.1%	
6 times and more	24.0%	6.8%	
What kind of entertainment do you choose in your spare time?			
Sleep	39.0%	31.8%	ns
Read	25.0%	33.1%	
Watch TV	45.0%	73.1%	
Surf the Internet and play games	55.0%	58.1%	
Shopping	5.0%	13.1%	
Sports such as running and playing ball	24.0%	20.8%	

ns: not significant. *P* < 0.05.

**Table 8:** Practices related to obesity among the population of Douala and Manjo according to BMI.

	Frequency (%)			*P*
	Normal weight BMI [18.5-25] (*n* = 547)	Overweight BMI [25-30] (*n* = 375)	Obesity BMI ≤ 30 (*n* = 290)	
How often do you eat breakfast each week?				
<2 days	29.3%	27.2%	22.4%	ns
2-3 days	32.5%	36.8%	33.1%	
3-5 days	25.8%	21.6%	23.8%	
>5 days	12.4%	14.4%	20.7%	
How many times have you had desserts in the past 7 days?				
None	30.7%	21.6%	22.8%	0.0123
1 time	22.9%	20.3%	14.5%	
2-6 times	22.1%	33.3%	23.8%	
Once every day	17.7%	17.3%	23.4%	
Twice a day or more	6.6%	7.5%	15.5%	
In the past 7 days, how many days did you eat fast food?				
None	46.1%	37.9%	27.2%	0.0045
1 day	20.3%	24.8%	21.8%	
2 days	17.9%	20.5%	23.1%	
3 days	9.5%	8.3%	17.2%	
4 days and more	6.2%	8.5%	10.7%	
In the past 7 days, how many days did you have at least one glass of milk/yogurt or soy milk?				
None	29.1%	27.5%	27.3%	ns
1 day	23.2%	22.1%	21.7%	
2 days	23.8%	26.1%	23.1%	
3 days	13.5%	17.1%	17.2%	
4 days and more	10.4%	7.2%	10.7%	
In the past 7 days, how many days did you eaten fruits/vegetables?				
None	12.6%	10.9%	10.3%	ns
1 day	17.7%	16.5%	10.7%	
2 days	27.1%	22.1%	25.2%	
3 days	22.3%	29.7%	26.9%	
4 days and more	20.3%	20.8%	26.9%	
In the past 7 days, how many days did you taken hot drinks (tea, tisanes...)?				
None	37.7%	29.4%	24.9%	0.0191
1 day	13.7%	12.0%	8.6%	
2 days	17.9%	18.4%	13.4%	
3 days	14.1%	15.7%	20.0%	
4 days and more	16.6%	24.5%	33.1%	
When you are in a bad mood, do you choose to eat a big meal?				
Yes	17.0%	22.7%	22.1%	ns
No	83.0%	77.3%	77.9%	
Do you choose foods based on their nutritional value in your daily meals?				
Always	14.1%	10.7%	9.7%	ns
Often	44.4%	39.7%	44.5%	
Rarely	28.9%	34.7%	26.2%	
Not at all	12.6%	14.9%	19.6%	
How many times do you do physical activity/exercise per month?				
None	16.1%	21.3%	25.5%	0.0138
1 time	11.9%	14.9%	21.0%	
2 times	14.6%	16.0%	18.7%	
3 times	10.6%	13.3%	8.6%	
4 times	12.6%	13.3%	9.0%	
5 times	6.2%	8.0%	3.1%	
6 times and more	28.0%	13.2%	14.1%	
What kind of entertainment do you choose in your spare time?				
Sleep	35.8%	37.6%	38.3%	ns
Read	25.8%	23.7%	33.4%	
Watch TV	44.8%	53.1%	64.1%	
Surf the Internet and play games	55.2%	55.7%	57.9%	
Shopping	5.9%	6.1%	10.3%	
Sports such as running and playing ball	27.4%	24.3%	13.4%	

ns: not significant. *P* < 0.05.

**Table 9:** Knowledge, attitude, and practice scores according to sociodemographic characteristics.

	Knowledge score	Attitude score	Practice score
Study site			
Douala (*n* = 892)	52.3 ± 22.6^a^	67.5 ± 20.0^b^	46.1 ± 19.2^a^
Manjo (*n* = 320)	66.7 ± 20.4^b^	63.2 ± 17.3^a^	52.1 ± 17.3^b^
*P*	<0.0001	0.0006	<0.0001
BMI			
Normal weight BMI [18.5-25] (*n* = 547)	53.1 ± 23.3^a^	66.6 ± 20.5^a,b^	47.5 ± 18.1^a^
Overweight BMI [25-30] (*n* = 375)	56.3 ± 21.7^a,b^	63.5 ± 19.9^a^	46.8 ± 16.7^a^
Obesity BMI ≤ 30 (*n* = 290)	59.7 ± 17.9^b^	67.3 ± 17.8^b^	47.7 ± 16.9^a^
*P*	0.0001	0.0202	ns
Gender			
Men (*n* = 510)	51.6 ± 28.1^a^	64.8 ± 20.4^a^	48.9 ± 16.8^b^
Women (*n* = 702)	53.7 ± 28.8^a^	68.1 ± 18.5^b^	46.5 ± 17.3^a^
*P*	ns	0.0012	0.0182
Age (years)			
≤30 (*n* = 755)	51.9 ± 29.4^a^	68.3 ± 20.2^b^	47.2 ± 17.2^a^
[30-50] (*n* = 401)	53.7 ± 27.6^a^	63.7 ± 20.6^a^	47.4 ± 17.7^a^
>50 (*n* = 56)	55.2 ± 28.3^a^	66.7 ± 17.8^b^	56.6 ± 17.8^b^
*P*	ns	0.0061	0.0002
Origin region			
Adamawa/north/far north (*n* = 65)	49.0 ± 25.5^a^	65.4 ± 18.4^a^	46.6 ± 16.6^a^
Centre/east/south (*n* = 271)	48.4 ± 28.1^a^	66.3 ± 21.0^a^	45.0 ± 18.7^a^
Littoral/southwest (*n* = 305)	53.9 ± 28.8^a^	65.7 ± 19.4^a^	46.8 ± 14.4^a^
West/northwest (*n* = 571)	51.9 ± 29.1^a^	66.6 ± 19.0^a^	48.0 ± 20.1^a^
*P*	ns	ns	ns
Education			
University studies (*n* = 728)	54.86 ± 30.00^b^	68.72 ± 20.07^b^	47.58 ± 17.90^b^
Secondary studies (*n* = 384)	52.62 ± 28.72^b^	63.07 ± 19.26^a^	46.11 ± 16.35^b^
Primary studies or none (*n* = 100)	42.22 ± 23.59^a^	59.93 ± 21.07^a^	42.94 ± 20.94^a^
*P*	0.0002	<0.0001	0.0062
Occupation			
Student (*n* = 659)	54.8 ± 27.1^b^	64.0 ± 18.0^a^	47.0 ± 20.1^b^
Unemployed (*n* = 179)	47.4 ± 28.0^a^	63.6 ± 19.2^a^	43.5 ± 18.6^a^
Employee (*n* = 142)	48.0 ± 29.3^a^	67.1 ± 18.1^b^	47.6 ± 17.9^b^
Self-employment (*n* = 232)	48.5 ± 27.9^a^	68.8 ± 20.3^b^	49.0 ± 20.6^b^
*P*	0.0270	0.0005	0.0025
Lifestyle			
Life with family (*n* = 1002)	54.0 ± 28.5^b^	66.57 ± 18.73^a^	46.57 ± 15.53^a^
Life alone or other (*n* = 210)	48.0 ± 29.3^a^	65.18 ± 20.78^a^	45.96 ± 17.13^a^
*P*	0.0056	ns	ns
Marital status			
Single/divorced/widow (*n* = 823)	49.2 ± 29.6^a^	66.5 ± 21.2^a^	45.9 ± 17.5^a^
Married (*n* = 389)	54.8 ± 28.1^b^	64.7 ± 20.9^a^	50.1 ± 17.6^b^
*P*	0.0009	ns	<0.0001
Religion			
Christian (*n* = 1078)	51.6 ± 28.7^a,b^	65.9 ± 19.7^a^	47.0 ± 17.3^a^
Muslim (*n* = 88)	46.2 ± 26.5^a^	62.6 ± 18.8^a^	48.7 ± 15.6^a,b^
Other (*n* = 46)	58.5 ± 29.4^b^	67.8 ± 21.2^a^	53.1 ± 16.5^b^
*P*	0.0496	ns	0.0402

ns: not significant. *P* < 0.05. For a given factor, scores in the same column with the same letter are not significantly different at *P* < 0.05.

**Table 10:** Pearson correlation coefficient between knowledge, attitude, and practice.

	Practice score	Attitude score	Knowledge score
Practice score	1		
Attitude score	0.253^ns^	1	
Knowledge score	0.578^∗∗^	0.195^ns^	1

ns: no significant correlation at *P* < 0.05. ^∗∗^Significant correlation at *P* < 0.01.

## Data Availability

All data used during this study are available upon request from the corresponding author.
